# Situated affective and social neuroscience

**DOI:** 10.3389/fnhum.2014.00547

**Published:** 2014-07-28

**Authors:** Agustin Ibanez, Sonja A. Kotz, Louise Barrett, Jorge Moll, Maria Ruz

**Affiliations:** ^1^Laboratory of Experimental Psychology and Neuroscience, Institute of Cognitive Neurology (INECO), Favaloro UniversityBuenos Aires, Argentina; ^2^National Scientific and Technical Research Council (CONICET)Buenos Aires, Argentina; ^3^UDP-INECO Foundation Core on Neuroscience, Diego Portales UniversitySantiago, Chile; ^4^Universidad Autónoma del CaribeBarranquilla, Colombia; ^5^Centre of Excellence in Cognition and its Disorders, Australian Research Council (ACR)Sydney, NSW, Australia; ^6^Cognitive Neuroscience and Experimental Psychology Section, School of Psychological Sciences, University of ManchesterManchester, UK; ^7^Department of Neuropsychology, Max Planck Institute of Human Cognitive and Brain SciencesLeipzig, Germany; ^8^Department of Psychology, University of LethbridgeLethbridge, AB, Canada; ^9^Cognitive and Behavioral Neuroscience Unit, D'Or Institute for Research and EducationRio de Janeiro, Brazil; ^10^Department of Experimental Psychology, Brain, Mind and Behavior Research Center, University of GranadaGranada, Spain

**Keywords:** social neuroscience, contextual social cognition, emotions, neuropsychiatry, embodied cognition, social behavior, emotion regulation, social decision making

This Research Topic features several papers tapping the situated nature of emotion and social cognition processes. The volume covers a broad scope of methodologies [behavioral assessment, functional magnetic resonance imaging (fMRI), structural neuroimaging, event-related potentials (ERPs), brain connectivity, and peripheral measures], populations (non-human animals, neurotypical participants, developmental studies, and neuropsychiatric and pathological conditions), and article types (original research, review papers, and opinion articles). Through this wide-ranging proposal, we introduce a fresh approach to the study of contextual effects in emotion and social cognition domains.

We report four levels of evidence. First, we present studies examining how cognitive and neural functions are influenced by basic affective processes (interoception, motivation and reward, emotional impulsiveness, and appraisal of violent stimuli). A second set of behavioral and neuroscientific studies addresses how performance is modulated by different emotional variables (categorical and dimensional approaches to emotion, language-as-context for emotion, emotional suppression of the attentional blink, and reappraisal effects on the up-regulation of emotions). The studies in our third selection deal with different influences in social cognition (SC) domains (human and non-human comparative studies, long-term effects of social and physical stress, developmental theory of mind, neural bases of passionate love for others, social decision making in normal and psychopathic participants, and frontal lobe contributions to psychosocial adaptation models). Finally, the fourth set of papers investigates the blending of social and emotion-related processes (valence and social salience in amygdala networks, emotional contributions to identification of genuine and faked social expressions, emotional predispositions and social decision making bias, valence of fairness and social decisions, structural neuroimaging of emotional and social impairments in neurodegenerative diseases, and subjective reactivity to emotional stimuli and their association with moral cognition). A brief summary of all these studies is offered in the following sections.

## Basic affective modulation

Body signals, especially of the interoceptive cardiac type, have been recently claimed to modulate emotion and decision-making processes. Leone et al. (2012) used chess decisions to analyze heart rate (HR) modulations in specific cognitive events. HR signals predicted the conception of a plan and the likelihood to blunder by fluctuations (e.g., performing random errors or bad moves). Such signals also reflected reactions, such as a blunder made by the opponent or fluctuations after a move. These data suggest that body signals are rich enough to reveal relevant episodes of inner decisions.

In another study, the affective motivational dimension of behavioral inhibition was assessed through the manipulation of reward magnitudes during a classical inhibitory task (Herrera et al., 2014). The effect of reward magnitude and context on behavioral inhibition in humans showed that dynamical behavioral inhibition depends on contextual parameters (reward magnitude modulation and initial reward history).

Torres et al. (2013) tested whether emotional and non-emotional dimensions of impulsiveness were differentially predictive of decision-making and addictive behavior in cocaine-dependent individuals (CDIs), pathological gamblers (PGs), and healthy controls. They used several instruments, including a Go/No-go paradigm assessed with ERPs and a delay-discounting task. Among the dimensions of trait impulsiveness, negative urgency was unique at independently covarying with gambling in PGs. Relative to these subjects, CDIs performed more poorly and showed ERP abnormalities. The effects of impulsiveness in negative emotion processing played a key role in decision-making and addiction.

Using fMRI, Porges and Decety (2013) evaluated the appraisal of violent stimuli and their relation with self-report measures of pleasure/displeasure. Participants watched video-clips depicting Mixed Martial Arts (MMA). Capoeira videos were used as a baseline. *Pleasurable* ratings of MMA predicted increased functional connectivity (FC) seeded in the nucleus accumbens, anterior cingulate cortex (ACC), and anterior insular cortex (AIC). These structures are related with positive/negative outcomes as well as feelings and somatic representations. Instead, *displeasure* ratings of MMA were related to increased FC among regions of the prefrontal cortex and superior parietal lobule (areas involved in cognitive control and executive attention). The results suggest that FC indexed the relationship between subjective feelings and anticipation of positive and negative outcomes.

## Emotional appraisal

Matsuda et al. (2013) tested the hypothesis that separate neural loci might intrinsically encode categorical and dimensional facial emotion perception. Participants were scanned with fMRI while they passively viewed emotional faces and performed unrelated tasks. Activity in the right fusiform face area (FFA) was dependent on the categorical ambiguity of facial expressions. The amygdala, insula, and medial prefrontal cortex evidenced dimensional (linear) processing, which correlated with physical changes in expressions. The results suggest that distinct neural loci process the physical and psychological aspects of facial emotion perception in a region-specific and implicit manner.

Herbert et al. (2013) used words as contextual cues for emotion processing in two ERP experiments. They focused on self- vs. sender-related emotional pronoun-noun pairs (e.g., my fear vs. his fear) as cues for emotional face processing. Participants performed automatic (Experiment 1) and intentional (Experiment 2) affect labeling tasks. ERP patterns varied as a function of the label's reference (self vs. sender) and the intentionality of the labeling task (Experiment 1 vs. Experiment 2). Emotion decoding from facial expressions was not fully determined by sensory facial information, but proved sensitive to contextual factors and the perceiver's experience. These findings support a differentiated view of language-as-context for emotion processing.

The study conducted by Kanske et al. (2013) evaluated whether the attentional blink effect in rapid serial visual presentations is modulated by the emotionality of the stimuli (emotional and neutral images depicting social scenes as target). To this end, the authors used ERP recordings and offline self-reports of empathy. The results revealed enhanced performance for emotional stimuli and increased P3 amplitudes, which correlated with individual differences in empathy. These data suggest that empathy is associated with enhanced emotional processing in social contexts, even during unconscious target detection.

Peng et al. (2013) examined description-based reappraisal effects on the up-regulation of positive emotions. They measured ERP fluctuations as Chinese participants viewed erotic and neutral images shown after either a neutral or positive description. Further data was obtained through self-reported ratings. The results demonstrate that description-based reappraisal significantly modulated the emotional experience and ERP responses to erotic as well as neutral images.

## Social cognition

The review by Van Den Bos et al. (2013) considers animal and human studies tapping the influence of social context on decision-making. From a causal and functional perspective, the authors advance methodological considerations to improve the experimental assessment of social factors in decision-making.

In a study with rats, Chaby et al. (2013) investigated how exposure to social and physical stress during adolescence affects adult decision-making, coping response, cognitive bias, and exploratory behavior. Compared to control animals, rats exposed to chronic unpredictable stress (e.g., isolation, crowding, cage tilt) evinced long-term behavioral and cognitive changes, including negative cognitive bias, altered coping response, and accelerated decision-making. The results showed that stress during adolescence has a long-term impact on behavior and cognition. The most salient effects concern ambiguous stimulus interpretation, behavioral response to adverse events, and decision-making strategies.

Calero et al. (2013) propose a novel approach to quantifying the scaling property of theory of mind (ToM). Focusing on children between 6- and 8-years-old, they consider a scaling complexity of skills and their modulation by varied factors, such as gender, number of siblings, and personality traits.

The meta-analysis by Juan et al. (2013) considers a decade worth of fMRI studies to identify differential brain areas and cortical networks involved in (i) passionate love for others and (ii) understanding the intention of others' actions. Thus, this approach goes beyond classical experimental studies regarding individuals as strictly isolated entities. Both overlapping and distinct cortical and subcortical regions were identified for intention and love, respectively. By targeting these brain regions in future research, scientists and clinicians could promote breakthroughs in the neuroscience of pair-bonding.

Radke et al. (2013) investigated fairness considerations in psychopathic and non-psychopathic offenders as well as healthy controls. In a modified Ultimatum Game (UG) involving opposing intentionality constraints (intentional vs. unintentional), unfair offers were paired with different unselected alternatives, thereby establishing the context of a proposal. Psychopathic offenders resembled healthy controls in their rejection pattern—i.e., they took the unselected alternative into account. In contrast, non-psychopathic delinquents failed to adjust their decisions to an offer's alternatives, suggesting stronger impairments in social decision-making. Crucially, the mechanisms and processes underlying rejection decisions might differ in both groups, particularly in terms of cognitive vs. emotional competencies.

In an ERP experiment, Moser et al. (2014) investigated the levels of processing at which positive and negative descriptions of other people bias social decision-making. Participants played a game in which they had to accept or reject economic offers. Other variables manipulated were the fairness of the assets' distribution, the offers' advantageousness, and the game context's uncertainty. Negative description of the interaction partner enhanced medial frontal negativity (MFN) in an additive manner with fairness evaluations. The description of the partner interacted with personal benefit considerations, showing that this positive or negative information biased the evaluation of offers only when they did not favor the participant. P300 amplitudes were enhanced by advantageous offers, suggesting their heightened motivational significance at later stages of processing. In all phases of the study, processing of the offer was increased in the certain, as compared to the uncertain, contexts. These results provide new evidence that decision-making is influenced by interpersonal information and considerations of one's own interests relative to those of others.

Finally, Huepe and Salas (2013) set forth a new conceptualization of the prefrontal cortex for psychosocial adaptation. Their review of the evidence suggests that cognitive functions related to this lobule include fluid intelligence (FI), SC, and perspective changing abilities (PCA). These domains are crucial in adapting to social contexts and solving problems in new situations. Moreover, they appear to depend on contextual keys, thus requiring flexibility—yet another function associated with the frontal lobe. The model proposed integrates these components (FI, SC, and PCA) as indicators of psychosocial adaptation in contexts of social vulnerability or impoverished social/cultural conditions.

## Contextual blending of social and emotion-related processes

Vrtička et al. (2013) assessed whether the human amygdala preferentially responds to both emotionally and socially significant information, and whether these factors might display interactive encoding properties. Through an fMRI study, they demonstrated that amygdala activation is (1) greater for neutral social vs. non-social information, (2) similar for positive and negative social images, and (3) sensitive to a valence effect (negative vs. positive) for non-social images. The valence × social content interaction was also found in the right fusiform gyrus, right anterior superior temporal gyrus, and medial orbitofrontal cortex. Overall, these findings suggest that valence and social contents possess distinct kinds of relevance that interact within the human amygdala and throughout a more extensive cortical network.

The ability to discriminate between felt and faked expressions is a crucial social skill. Manera et al. (2013) investigated whether individual differences in smile authenticity recognition are explained by distinct predispositions to experience other people's emotions (susceptibility to emotional contagion). Susceptibility to emotional contagion for negative emotions increased smile authenticity detection. Instead, susceptibility to emotional contagion for positive emotions worsened detection performance, because it led to categorize most faked smiles as sincere. It follows that susceptibility to emotional contagion plays a key role in complex social emotions.

The study by Klapwijk (2013) examined the effects of three different emotional responses (anger, disappointment, and happiness) on social decision-making in adolescents. In a version of the Dictator Game, unfair offers by the participants received emotional responses from peers. Relative to angry and happy reactions, expressions of disappointment prompted more generous offers. Older adolescents were better than younger adolescents at differentiating among the three emotions. In addition, individual differences in social value orientation played a role in decisions after happy reactions to unfair offers. Thus, adolescents take into account the emotions of their peers when making social decisions and are affected by social value orientation and age.

Couto et al. (2013) report selective behavioral impairments of face recognition, emotion recognition, and ToM in patients with behavioral variant frontotemporal dementia (bvFTD) and progressive non-fluent aphasia (PNFA). Voxel-based morphometry revealed fronto-temporo-insular atrophy in both patient groups. SC deficits were differentially associated to fronto-insular-temporal atrophy in bvFTD and PNFA, respectively. While SC impairments were similar in both groups, they seem to reflect intrinsic ToM affectation in bvFTD and more basic deficits (face and emotion recognition) in PNFA.

Carmona-Perera et al. (2013) examined subjective reactivity to emotional stimuli and its possible association with moral decision-making. Healthy adult participants responded to a set of moral and non-moral dilemmas. The researchers focused on emotional experience in valence, arousal, and dominance dimensions in response to different types of pictures (neutral, pleasant, unpleasant non-moral, and unpleasant moral). Significant correlations emerged between less unpleasantness to negative stimuli, more pleasantness to positive stimuli, and a higher proportion of utilitarian choices. Also, a positive association was found between higher arousal ratings to negative moral laden pictures and more utilitarian choices. Low dominance was associated with greater perceived difficulty over moral judgment. These results evidenced a contextual role of emotional experience in moral choice.

## Conclusions

Despite the diversity of their topics, research questions, and methodologies, most of these studies highlight the contextual situatedness of emotional and social cognition processes (Garrido-Vasquez et al., 2011; Ruz and Tudela, 2011; Ibanez and Manes, 2012; Melloni et al., 2014). Moreover, they provide new evidence for the interaction among low and high-level cognition, emotion, and social domains (Moll and Schulkin, 2009; Pessoa, 2009; Alguacil et al., 2013; Ibanez et al., 2013, 2014; Ruz et al., 2013; Baez et al., 2014b). In the same vein, part of the evidence presented shows that our emotional arousal biases our decisions in the social world (Beauregard, 2007; Heatherton, 2011). More generally, this Research Topic indicates that a brain network approach to social and emotional processes (Moll et al., 2005, 2008; Kennedy and Adolphs, 2012; Baez et al., 2014a) seems more adequate than simple approximations ascribing such complex domains to a single region. This integrated approach to embedded emotional and social processes provides exciting new avenues into the growing field of social neuroscience.

### Conflict of interest statement

The authors declare that the research was conducted in the absence of any commercial or financial relationships that could be construed as a potential conflict of interest.

## References

[B1] Alguacil S. Tudela P. Ruz M. (2013). Cognitive and affective control in a flanker word task: common and dissociable brain mechanisms. Neuropsychologia 51, 1663–1672 10.1016/j.neuropsychologia.2013.05.020 23747603

[B2] Baez S. Couto B. Torralva T. Sposato L. Huepe D. Montañes P. (2014a). Comparing the moral judgments of frontotemporal dementia and frontal stroke patients. JAMA Neurol. 10.1001/jamaneurol.2014.347 25047907

[B3] Baez S. Marengo J. Perez A. Huepe D. Font F. G. Rial V. (2014b). Theory of mind and its relationship with executive functions and emotion recognition in borderline personality disorder. J. Neuropsychol. [Epub ahead of print]. 10.1111/jnp.12046 24766847

[B4] Beauregard M. (2007). Mind does really matter: evidence from neuroimaging studies of emotional self-regulation, psychotherapy, and placebo effect. Prog. Neurobiol. 81, 218–236 10.1016/j.pneurobio.2007.01.005 17349730

[B5] Calero C. I. Salles A. Semelman M. Sigman M. (2013). Age and gender dependent development of Theory of Mind in 6- to 8-years old children. Front. Hum. Neurosci. 7:281 10.3389/fnhum.2013.00281 23785326 PMC3683618

[B6] Carmona-Perera M. Martí-García C. Pérez-García M. Verdejo-García A. (2013). Valence of emotions and moral decision-making: increased pleasantness to pleasant images and decreased unpleasantness to unpleasant images are associated with utilitarian choices in healthy adults. Front. Hum. Neurosci. 7:626 10.3389/fnhum.2013.00626 24133433 PMC3783947

[B7] Chaby L. E. Cavigelli S. A. White A. Wang K. Braithwaite V. A. (2013). Long-term changes in cognitive bias and coping response as a result of chronic unpredictable stress during adolescence. Front. Hum. Neurosci. 7:328 10.3389/fnhum.2013.00328 23847501 PMC3701140

[B8] Couto B. Manes F. Montañés P. Matallana D. Reyes P. Velasquez M. (2013). Structural neuroimaging of social cognition in progressive non-fluent aphasia and behavioral variant of frontotemporal dementia. Front. Hum. Neurosci. 7:467 10.3389/fnhum.2013.00467 23966929 PMC3744869

[B9] Garrido-Vasquez P. Jessen S. Kotz S. A. (2011). Perception of emotion in psychiatric disorders: on the possible role of task, dynamics, and multimodality. Soc. Neurosci. 6, 515–536 10.1080/17470919.2011.620771 21961831

[B10] Heatherton T. F. (2011). Neuroscience of self and self-regulation. Annu. Rev. Psychol. 62, 363–390 10.1146/annurev.psych.121208.131616 21126181 PMC3056504

[B11] Herbert C. Sfärlea A. Blumenthal T. (2013). Your emotion or mine: labeling feelings alters emotional face perception—an ERP study on automatic and intentional affect labeling. Front. Hum. Neurosci. 7:378 10.3389/fnhum.2013.00378 23888134 PMC3719026

[B12] Herrera P. M. Speranza M. Hampshire A. Bekinschtein T. N. A. (2014). Monetary rewards modulate inhibitory control. Front. Hum. Neurosci. 8:257 10.3389/fnhum.2014.00257 24860469 PMC4026705

[B13] Huepe D. Salas N. (2013). Fluid intelligence, social cognition, and perspective changing abilities as pointers of psychosocial adaptation. Front. Hum. Neurosci. 7:287 10.3389/fnhum.2013.00287 23785329 PMC3684846

[B14] Ibanez A. Aguado J. Baez S. Huepe D. Lopez V. Ortega R. (2014). From neural signatures of emotional modulation to social cognition: individual differences in healthy volunteers and psychiatric participants. Soc. Cogn. Affect. Neurosci. 9, 939–950 10.1093/scan/nst067 23685775 PMC4090956

[B15] Ibanez A. Huepe D. Gempp R. Gutiérrez V. Rivera-Rei A. Toledo M. (2013). Empathy, sex and fluid intelligence as predictors of theory of mind. Pers. Individ. Dif. 54, 616–621 10.1016/j.paid.2012.11.022

[B16] Ibanez A. Manes F. (2012). Contextual social cognition and the behavioral variant of frontotemporal dementia. Neurology 78, 1354–1362 10.1212/WNL.0b013e3182518375 22529204 PMC3335455

[B17] Juan E. Frum C. Bianchi-Demicheli F. Wang Y.-W. Lewis J. W. Cacioppo S. (2013). Beyond human intentions and emotions. Front. Hum. Neurosci. 7:99 10.3389/fnhum.2013.00099 23543838 PMC3608908

[B18] Kanske P. Schönfelder S. Wessa M. (2013). Emotional modulation of the attentional blink and the relation to interpersonal reactivity. Front. Hum. Neurosci. 7:641 10.3389/fnhum.2013.00641 24130525 PMC3795393

[B19] Kennedy D. P. Adolphs R. (2012). The social brain in psychiatric and neurological disorders. Trends Cogn. Sci. 16, 559–572 10.1016/j.tics.2012.09.006 23047070 PMC3606817

[B20] Klapwijk E. (2013). Emotional reactions of peers influence decisions about fairness in adolescence. Front. Hum. Neurosci. 7:745 10.3389/fnhum.2013.00745 24282399 PMC3824368

[B21] Leone M. J. Petroni A. Fernandez Slezak D. Sigman M. (2012). The tell-tale heart: heart rate fluctuations index objective and subjective events during a game of chess. Front. Hum. Neurosci. 6:273 10.3389/fnhum.2012.00273 23060777 PMC3465955

[B22] Manera V. Grandi E. Colle L. (2013). Susceptibility to emotional contagion for negative emotions improves detection of smile authenticity. Front. Hum. Neurosci. 7:6 10.3389/fnhum.2013.00006 23508036 PMC3600526

[B23] Matsuda Y.-T. Fujimura T. Katahira K. Okada M. Ueno K. Cheng K. (2013). The implicit processing of categorical and dimensional strategies: an fMRI study of facial emotion perception. Front. Hum. Neurosci. 7:551 10.3389/fnhum.2013.00551 24133426 PMC3783839

[B24] Melloni M. Lopez V. Ibanez A. (2014). Empathy and contextual social cognition. Cogn. Affect. Behav. Neurosci. 14, 407–425 10.3758/s13415-013-0205-3 23955101

[B25] Moll J. De Oliveira-Souza R. Zahn R. (2008). The neural basis of moral cognition: sentiments, concepts, and values. Ann. N.Y. Acad. Sci. 1124, 161–180 10.1196/annals.1440.005 18400930

[B26] Moll J. Schulkin J. (2009). Social attachment and aversion in human moral cognition. Neurosci. Biobehav. Rev. 33, 456–465 10.1016/j.neubiorev.2008.12.001 19126412

[B27] Moll J. Zahn R. De Oliveira-Souza R. Krueger F. Grafman J. (2005). Opinion: the neural basis of human moral cognition. Nat. Rev. Neurosci. 6, 799–809 10.1038/nrn1768 16276356

[B28] Moser A. Gaertig C. Ruz M. (2014). Social information and personal interests modulate neural activity during economic decision-making. Front. Hum. Neurosci. 8:31 10.3389/fnhum.2014.00031 24567708 PMC3915773

[B29] Peng J. Qu C. Gu R. Luo Y.-J. (2013). Description-based reappraisal regulate the emotion induced by erotic and neutral images in a Chinese population. Front. Hum. Neurosci. 6:355 10.3389/fnhum.2012.00355 23335894 PMC3541519

[B30] Pessoa L. (2009). How do emotion and motivation direct executive control? Trends Cogn. Sci. 13, 160–166 10.1016/j.tics.2009.01.006 19285913 PMC2773442

[B31] Porges E. C. Decety J. (2013). Violence as a source of pleasure or displeasure is associated with specific functional connectivity with the nucleus accumbens. Front. Hum. Neurosci. 7:447 10.3389/fnhum.2013.00447 23964226 PMC3741555

[B32] Radke S. Brazil I. A. Scheper I. Bulten B. H. De Bruijn E. R. A. (2013). Unfair offers, unfair offenders? Fairness considerations in incarcerated individuals with and without psychopathy. Front. Hum. Neurosci. 7:406 10.3389/fnhum.2013.00406 23898257 PMC3724121

[B33] Ruz M. Madrid E. Tudela P. (2013). Interactions between perceived emotions and executive attention in an interpersonal game. Soc. Cogn. Affect. Neurosci. 8, 838–844 10.1093/scan/nss080 22842814 PMC3791075

[B34] Ruz M. Tudela P. (2011). Emotional conflict in interpersonal interactions. Neuroimage 54, 1685–1691 10.1016/j.neuroimage.2010.08.039 20736070

[B35] Torres A. Catena A. Megías A. Maldonado A. Cándido A. Verdejo-García A. (2013). Emotional and non-emotional pathways to impulsive behavior and addiction. Front. Hum. Neurosci. 7:43 10.3389/fnhum.2013.00043 23441001 PMC3578351

[B36] Van Den Bos R. Jolles J. W. Homberg J. R. (2013). Social modulation of decision-making: a cross-species review. Front. Hum. Neurosci. 7:301 10.3389/fnhum.2013.00301 23805092 PMC3693511

[B37] Vrtička P. Sander D. Vuilleumier P. (2013). Lateralized interactive social content and valence processing within the human amygdala. Front. Hum. Neurosci. 6:358 10.3389/fnhum.2012.00358 23346054 PMC3548516

